# Supplementary material for: CRISPR-Cas9 ribonucleoprotein-mediated co-editing and counterselection in the rice blast fungus
Source: Sci Rep. 2018 Sep 25;8:14355. doi: 10.1038/s41598-018-32702-w (PMC6156577; doi:10.1038/s41598-018-32702-w)
Supplement: Supplementary file 1 — Supplementary Information [file 41598_2018_32702_MOESM1_ESM.docx]

**SUPPLEMENTARY MATERIALS**

**CRISPR-Cas9 ribonucleoprotein-mediated co-editing and counterselection in the rice blast fungus**

Andrew J. Foster, Magdalena Martin-Urdiroz, Xia Yan, Sabrina Wright, Darren M. Soanes and Nicholas J. Talbot

***
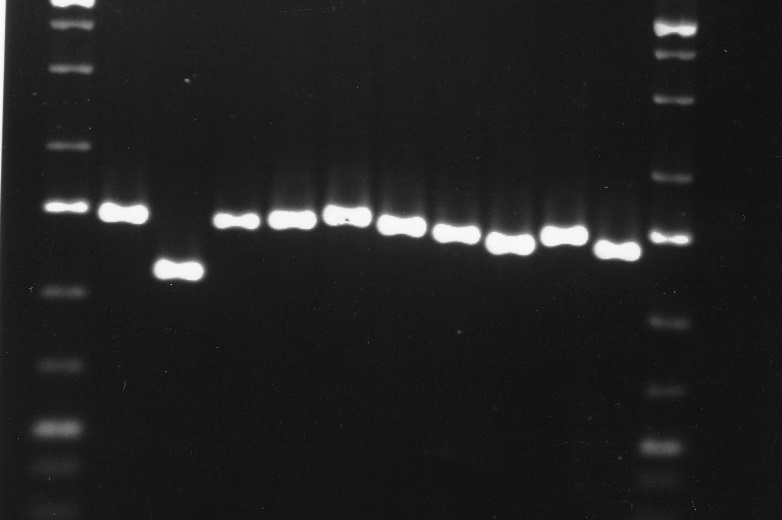
***

**Figure S1. Full length gel image for the gel shown in Figure 3 showing range of mutations generated using RNP without donor DNA targeting *ALB1*.**  Gel electrophoresis of the PCR products generated using the genomic DNA of the purified albino regenerants and primers PKS-ck-F and PKS-ck-R which flank the *ALB1*-targeting RNP-CRISPR-Cas9 genomic target sequence showing visible variation in product size.

***ALB1* the native locus at the target sequence**

**GCCACAAGCTCCCTCACCAAGGCCACGGCCATCCCGGTCTACGGCCCGTACCACGCCCCCCACCTCCACCGCCCTGAGGATGTCGACAACATCCTGGGCCTCAACAACCCCGAGATGATTGACACCTTCCTCAACACCAAGCCCCGCTCCTCCGTCATGTCC**

**Alb-(1)top (donor DNA to introduce a stop codon within the genomic target sequence)**

**CTCCCTCACCAAGGCCACGGCCATCCCGGTCTACGGCCCGTAGCACGCCCCCCACCTCCACCGCCCTGAGGATGTCGAC**

**Alb-(1)bot (this oligo is annealled to Alb1-(1)top to form a dsDNA donor)**

**GTCGACATCCTCAGGGCGGTGGAGGTGGGGGGCGTGCTACGGGCCGTAGACCGGGATGGCCGTGGCCTTGGTGAGGGAG**

**Alb1-(2)top (donor DNA to introduce a stop codon at a distance from the genomic target sequence)**

**CTCCCTCACCAAGGCCACGGCCATCCCGGTCTACGGCCCGTACCACGCCCCCCACCTCCACCGCCCTTAGGATGTCGAC**

**Alb-(2)bot (this oligo is annealled to Alb1-(1)top to form a dsDNA donor)**

**GTCGACATCCTAAGGGCGGTGGAGGTGGGGGGCGTGGTACGGGCCGTAGACCGGGATGGCCGTGGCCTTGGTGAGGGAG**

**Figure S2. Oligonucleotide donors used in RNP-CRISPR-Cas9 co-editing experiments to introduce a premature stop codon in *ALB1*.**

The oligonucleotide donors used to target *ALB1* by introducing a premature stop codon are illustrated together with the genomic target sequence for the *ALB1* targeting RNP-CRISPR-Cas9 complex. In the case of donor 1-1 the edit is within the genomic target sequence whereas in the case of donor 1-2 the edit is located 40 bp away from the PAM site used. DsDNA donors were generated by mix equimolar concentrations of the ‘top’ and ‘bot’ oligo together heating at 95oC for 5 mins and then cooling on the bench top. 2 μg of annealed oligo was transformed together with the *ALB1* targeting RNP


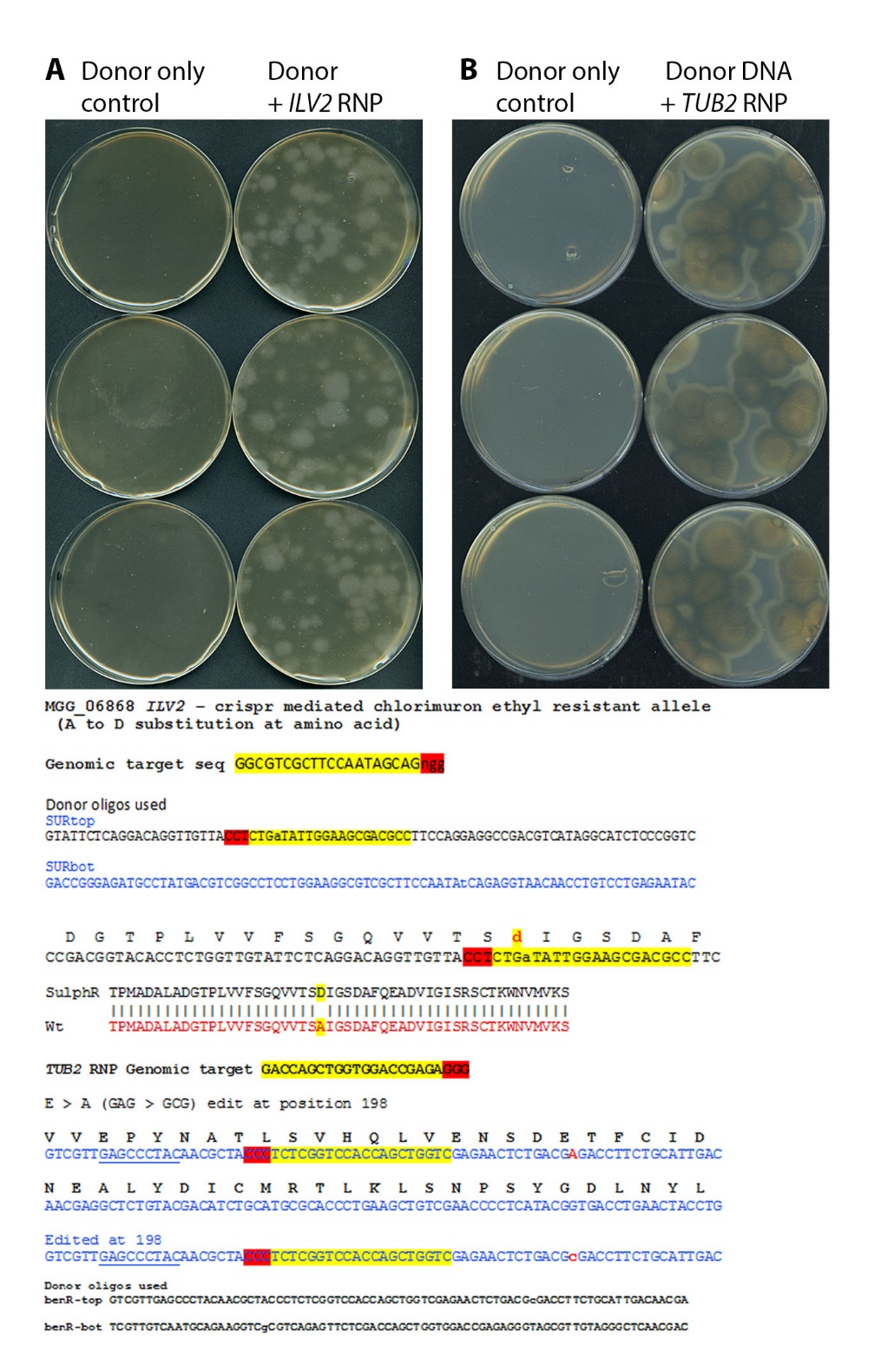


**C**

**D**

**Figure S3. Oligonucleotide donors used in RNP-CRISPR-Cas9 co-editing experiments targeting *TUB2* or *ILV2* and plates showing the selection procedure and the results of the transformation with the appropriate RNP complex.**

**a.** Transformation plates are shown for the *ILV2*-targeting RNP transformed together with the donor DNAs shown in **c.** **b.** Transformation plates are shown for the *TUB2*-targeting RNP transformed together with the donor DNAs shown in **d**. In **c** and **d** the design of the oligonucleotides conferring resistance to sulfonylurea and benomyl respectively is illustrated with their location relative to the genomic target sequence for the respective RNP complex.

**Table S1. Raw data for the transformation numbers presented in Fig. 1a using constructs expected to stably and constitutively express Cas9**

| Number of  transformants | Empty vector^a^ | *ALB1* targeting  vector^b^ | *PKS1* targeting  vector^c^ |  |  |
| --- | --- | --- | --- | --- | --- |
| Experiment 1 | 67 | 2 | 2 |  |  |
| Experiment 2 | 52 | 1 | 6 |  |  |
| Experiment 3 | 64 | 0 | 1 |  |  |
| Experiment 4 | 83 | 3 | 1 |  |  |

*Agrobacterium* compatible vectors containing the Cas9-NLS encoding gene under the control of the TrpC promoter and terminator were introduced into Guy 11 using *Agrobacterium*-mediated transformation. Transformant numbers were assessed after 7 days on selective medium (transformants were subsequently sub-cultured for assessment of pigmentation after growth on CM). None of the transformants isolated exhibited pigmentation defects. Three different versions (including the version functional in *N. crassa* (Matsu-Ura et al., 2015) shown used here) of the Cas9-NLS-encoding gene codon optimised for different fungi were tested in all every time with the same result that very few transformants were generated and none of these exhibited pigmentation alterations (data not shown).

**Table S2. Summary of transformation experiments targeting *ALB1* and *RSY1* using the RNP-Crispr method and donor only controls**

| RNPs donors used | Number of transformants | Number that are albino/rosy | Efficiency (%) | |  |  |
| --- | --- | --- | --- | --- | --- | --- |
| *ALB1* Donor DNA + *ALB1* -RNP | 4 | 4 | | 100 % |  |  |
| Control: *ALB1* Donor DNA only | 2 | 0 | | 0 % |  |  |
| *ALB1* Donor DNA + *ALB1* RNP | 112 | 96 | | 86 % |  |  |
| *ALB1* Donor DNA only | 64 | 3 | | 5% |  |  |
| *ALB1* Donor DNA + *ALB1* RNP | 16 | 8 | | 50 % |  |  |
| *ALB1* Donor DNA only | 11 | 1 | | 9 % |  |  |
| *ALB1* Donor DNA + *ALB1* RNP | 76 | 54 | | 71 % |  |  |
| *ALB1* Donor DNA only | 43 | 5 | | 12 % |  |  |
| *ALB1* Donor DNA + *ALB1* RNP | 86 | 69 | | 80 % |  |  |
| Donor DNA only | 48 | 7 | | 14 % |  |  |
| *RSY1* Donor DNA + *RSY1* RNP | 20 | 20 | | 100 % |  |  |
| Control: *RSY1* Donor DNA only | 3 | 0 | | 0 % |  |  |
| *RSY1* Donor DNA + *RSY1* RNP | 38 | 37 | | 97.5 % |  |  |
| Control: *RSY1* Donor DNA only | 8 | 1 | | 12.5 % |  |  |
| *RSY1* Donor DNA + *RSY1* RNP | 12 | 12 | | 100 % |  |  |
| Control: *RSY1* Donor DNA only | 13 | 1 | | 8 % |  |  |
| *RSY1* Donor DNA + *RSY1* RNP | 61 | 60 | | 98 % |  |  |
| Control: *RSY1* Donor DNA only | 47 | 5 | | 11 % |  |  |

The number of transformants for individual transformation experiments using *ALB1*-trageting-RNP-CRISPR-Cas9 or *RSY1*-targeting RNP-CRISPR-Cas9 complexes are shown. Also shown are donor only controls which show the efficiency obtained in the absence of the RNP complex and illustrate the efficiency of gene targeting which would be obtained in a ‘traditional’ gene deletion experiment.

**Table S3. Summary of transformation experiments targeting *ALB1* using the RNP-Crispr method and PCR amplified donor DNAs with 40 bp or 30 bp regions of homology to *ALB1* at either end**

| RNPs | Donor | Number of transformants | No. of albino mutants | |  | Efficiency (%) |
| --- | --- | --- | --- | --- | --- | --- |
| *ALB1* -RNP | 40 bp flanks | 11 | | 8 |  | 73 % |
| None | 40 bp flanks | 5 | | 0 |  | 0 % |
| *ALB1* RNP | 40 bp flanks | 48 | | 27 |  | 56 % |
| None | 40 bp flanks | 37 | | 0 |  | 0% |
| *ALB1* RNP | 40 bp flanks | 16 | | 10 |  | 63 % |
| None | 40 bp flanks | 22 | | 0 |  | 0 % |
| *ALB1*-RNP | 30 bp flanks | 29 | | 17 |  | 59 % |
| None | 30 bp flanks | 12 | | 0 |  | 0 % |
| *ALB1*-RNP | 30 bp flanks | 50 | | 28 |  | 56 % |
| None | 30 bp flanks | 59 | | 0 |  | 0 % |
| *ALB1*-RNP | 30 bp flanks | 24 | | 11 |  | 46 % |
| None | 30 bp flanks | 21 | | 0 |  | 0 % |

The number of transformants for individual transformation experiments using *ALB1*-targeting-RNP-CRISPR-Cas9 complexes in combination with the PCR generated donor DNA where a repair template with either 30 bp or 40 bp *ALB1* homologous regions flank the *BAR* gene conferring glufosinate- ammonium (basta) resistance. Also shown are donor only controls which show the efficiency obtained in the absence of the RNP complex.

**Table S4. Co-editing experiments based on selection for carboxin, sulfonylurea or benomyl resistance**

| **RNPs** | **Donors** | **Strain** | **No. examined** | **No. of *alb1* mutants** | **No with correct edit** | **Efficiency (%)** |
| --- | --- | --- | --- | --- | --- | --- |
| *SDI1* + *ALB1* | *SDI1R* (80 bp oligos) + *ALB1 STOP inside target* | Guy 11 | 191 | 5 | 3 | 1.6% |
| *SDI1* + *ALB1* | As above | Guy 11 | 276 | 3 | 3 | 1.1 % |
| *SDI1* + *ALB1* | As above | Guy 11 | 339 | 4 | 3 | 0.9 % |
| *SDI1* + *ALB1* | As above | Guy 11 | 369 | 11 | 6 | 1.6 % |
| *SDI1* + *ALB1* | As above | Δ*Ku70* | 37 | 3 | 3 | 12.3% |
| *SDI1* + *ALB1* | As above | Δ*Ku70* | 23 | 0 | 0 | 0 % |
| *SDI1* + *ALB1* | As above | Δ*Ku70* | 29 | 1 | 1 | 3.4 % |
| *SDI1* + *ALB1* | As above | Δ*Ku70* | 45 | 3 | 3 | 6.7 % |
| *SDI1* + *ALB1* | SDI1 carboxinR(80 bp oligos) *ALB1* STOP outside target | Guy 11 | 275 | 5 | 3 | 1.1% |
| *SDI1* + *ALB1* | As above | Guy 11 | 241 | 2 | 2 | 0.8 % |
| *SDI1* + *ALB1* | As above | Guy 11 | 298 | 2 | 2 | 0.7 % |
| *SDI1* + *ALB1* | As above | Guy 11 | 351 | 4 | 2 | 0.5 % |
| *TUB2* + *ILV2* | SulfonylureaR+ BenomylR | Guy 11 | 259 | 2 | 2 | 0.7 % |
| *TUB2* + *ILV2* | SulfonylureaR + BenomylR | Guy 11 | 384 | 4 | 4 | 1.0 % |
| *TUB2* + *ILV2* | SulfonylureaR + BenomylR | Guy 11 | 256 | 4 | 4 | 1.5 % |

The number of transformants generated in co-editing experiments using Guy 11 or the *Ku70*^-^ strain (23) are shown. Transformants were picked before any pigmentation was apparent. Co-editing-based mutation of *ALB1* used the oligonucleotides shown in Fig. S1 where the edit is either within the genomic target sequence (donor 1-1) of 40 bp distant to the PAM site (donor 1-2). Because *ALB1* mutants might also contain indels rather than integrations of the donors all albino strains were also checked by PCR amplification of DNA surrounding the target site and the efficiency is defined as the total number of transformants from those picked that have the correct integration of the donor DNA confirm by sequencing. In the case of co-editing with the *TUB2* and *ILV2* targeting CRISPR-Cas9-RNPs sequencing was only performed to check that the desired edits were present. In the case of *TUB2* indels can be expect to lead to loss of viability so that the efficiency was assessed directly as the proportion of the sulfonylurea resistant transformants that were also benomyl resistant. *TUB2* is MGG_00604 and *ILV2* is MGG_06868

**Table S5. Summary of genome reads and assemblies used in the current study**

| Strain | Number of reads^a^ | Size of assembly (bp)^b^ | Number of scaffolds^b^ | N50 contig length (bp)^b^ | Mean Coverage^c^ |
| --- | --- | --- | --- | --- | --- |
| Alb1_3 | 25,418,446 | 38,054,642 | 3,676 | 49,277 | 132.1x |
| Alb1_6 | 10,549,692 | 38,356,752 | 1,900 | 78,092 | 52.9x |
| R1 | 30,222,537 | 38,313,674 | 3,985 | 53,480 | 158.7x |
| R2 | 26,471,457 | 38,078,197 | 3,727 | 53,653 | 139.8x |

^a^125 base paired-end

^b^ Genome assembled ‘de novo’ using SPAdes 3.11.0 (46)

^c^ mapped against reference genome 70-15 (47) using BWA (48)

To determine whether off target mutations had occurred in the sequenced mutants, the two *alb1* mutants generated by CRISPR-Cas9-RNP and the two strains that had been through the transformation procedure and regenerated but not exposed to RNP complexes were sequenced. Sequences were generated using an Illumina HiSeq 2500 with standard reagents and protocols producing 125 base paired-end reads. Reads were filtered using the fastq-mcf program from the ea-utils package, ea-utils (45): "Command-line tools for processing biological sequencing data"; https://github.com/ExpressionAnalysis/ea-utils). Genomes were assembled ‘de novo’ using SPAdes 3.11.0 (46).

**Table S6. Sequences of the oligonucleotides used in the current study**

| Name | Sequence |
| --- | --- |
| ALB1-for-EcoRI | Primers used in construction of vectors  ctaatcaatcgaattcGGATTCCTCGCCGAGTTCTAC |
| ALB1-rev-SpeI | ctatacaactagtTCACGCCAGTCATCGGAAGTC |
| ALB1-for | GGATTCCTCGCCGAGTTCTAC |
| ALB1-rev | CATGCCAGGCTTCTCAATCCAG |
| RSY-donor-f | gccgactttgagccaagaagc |
| RSY-donor-r | cccggtgcaagccttacctag |
| Cas9-recom-f | GGATAATTCAATAGGCCAACAACACGTTGACGGAGAAGATGATATTGAAGGAGCATTTTTGG |
| Cas9-recom-r | GGTTCAATTGCGATTCATGTGGTGCGAGCAGTCTTTGTCCTCTAGAAAGAAGGATTACCTCTAAAC |
| PKS1gRNA-f | TCTTTGAAAAGATAATGTATGATTATGCTTTCACTC |
| PKS1gRNA-r | CTTATTTTAACTTGCTATTTCTAGCTCTAAAACCTCGTTTAGTTGTTGCAGTGGATCATTTATCTTTCACTGCGGAGAAGTTTCGAACGCC |
| SDH1-sg-r | CTTATTTTAACTTGCTATTTCTAGCTCTAAAACGAGCTTGTCGAGGAAGGAGCGATCATTTATCTTTCACTGCGGAGAAGTTTCGAACGCC |
| gRNAto315Cas-R | GTTCAATTGCGATTCATGTGGTGCGAGCAGTCTTTGTCTCTTTGAAAAGATAATGTATGATTATGCTTTCACTC |
| sgRNA-rec-R | GAGCAGTCTTTGTCCTCTAGAAAGAAGGATTACCTCTAAACAGACATAAAAAACAAAAAAAGCACCACCGACTCGG |
|  | Primers used as templates for sgRNA synthesis |
| sgSDI1 | TTCTAATACGACTCACTATAGAATGGTGTGGCAACGGTACGTTTTAGAGCTAGA |
| SgTUB2 | TTCTAATACGACTCACTATAGACCAGCTGGTGGACCGAGAGTTTTAGAGCTAGA |
| sgILV2 | TTCTAATACGACTCACTATAGGCGTCGCTTCCAATAGCAGGTTTTAGAGCTAGA |
| Sg-SEP5-n-gfp | TTCTAATACGACTCACTATAGAACTTGAAAGGAATTTGGTGTTTTAGAGCTAGA |
| sgSEP5ts | TTCTAATACGACTCACTATAGTCTGTGGAGCCTCAGGAACGTTTTAGAGCTAGA |
| sgSDH1  sgPKS-2 | TTCTAATACGACTCACTATAGCTCCTTCCTCGACAAGCTCGTTTTAGAGCTAGA  TTCTAATACGACTCACTATAGCGTGGTACGGGCCGTAGACGTTTTAGAGCTAGA  Primers used for PCR amplification of donor DNAs with BAR or GFP |
| ALB-BARkoF | GCCGCCACAAGCTCCCTCACCAAGGCCACGGCCATCCCGGTCGTCGACAGAAGATGATATTGAAGGAGC |
| ALB-BARkoR | GCAGAGCCTCTTGCATGAAACCTTGAAGCTGCTTGGCCTGGTCGACCTAAATCTCGGTGACGG |
| ALB-BARkoS-f | AAGCTCCCTCACCAAGGCCACGGCCATCCCGGTCGTCGACAGAAGATGATATTGAAGGAGC |
| ALB-BARkos-r | TTGCATGAAACCTTGAAGCTGCTTGGCCTGGTCGACCTAAATCTCGGTGACGG |
| SEP5-N-GFP-f | CAACATCGATCTAACCAACCAAATTCCTTTCAAGTTCAACACATGGTGAGCAAGGGCGAG |
| SEP5-N-GFP-r | gaaacggtccagaaggttgcggtcaacgtacGGCGGGAAACGACATCTTGTACAGCTCGTCCATGCC  Primers used to anneal together to form dsDNA donor DNAs |
| Alb-(1)top | CTCCCTCACCAAGGCCACGGCCATCCCGGTCTACGGCCCGTAGCACGCCCCCCACCTCCACCGCCCTGAGGATGTCGAC |
| Alb-(1)bot | GTCGACATCCTCAGGGCGGTGGAGGTGGGGGGCGTGCTACGGGCCGTAGACCGGGATGGCCGTGGCCTTGGTGAGGGAG |
| Alb1-(2)top | CTCCCTCACCAAGGCCACGGCCATCCCGGTCTACGGCCCGTACCACGCCCCCCACCTCCACCGCCCTTAGGATGTCGAC |
| Alb-(2)bot | GTCGACATCCTAAGGGCGGTGGAGGTGGGGGGCGTGGTACGGGCCGTAGACCGGGATGGCCGTGGCCTTGGTGAGGGAG |
| SDI1-80bp-top | CGCCTGGCCCTTGACAACTCCATGAGCCTGTACCGTTGCCTCACCATTCTTAACTGCACAAGGACCTGCCCGAAGGGCCT |
| SDI1-80bp-bot | AGGCCCTTCGGGCAGGTCCTTGTGCAGTTAAGAATGGTGAGGCAACGGTACAGGCTCATGGAGTTGTCAAGGGCCAGGCG |
| SDI1-60bp-top | TTGACAACTCCATGAGCCTGTACCGTTGCCTCACCATTCTTAACTGCACAAGGACCTGCC |
| SDI1-60bp-bot | GGCAGGTCCTTGTGCAGTTAAGAATGGTGAGGCAACGGTACAGGCTCATGGAGTTGTCAA |
| SDI1-50bp-top | TTGACAACTCCATGAGCCTGTACCGTTGCCTCACCATTCTTAACTGCACA |
| SDI1-50bp-bot | TGTGCAGTTAAGAATGGTGAGGCAACGGTACAGGCTCATGGAGTTGTCAA |
| SDI1-40bp-top | TTGACAACTCCATGAGCCTGTACCGTTGCCTCACCATTCT |
| SDI1-40bp-bot | AGAATGGTGAGGCAACGGTACAGGCTCATGGAGTTGTCAA |
| SDI1-30bp-top | AACTCCATGAGCCTGTACCGTTGCCTCACC |
| SDI1-30bp-bot | GGTGAGGCAACGGTACAGGCTCATGGAGTT |
| SDI1-20bp-top | GAGCCTGTACCGTTGCCTCA |
| SDI1-20bp-bot | TGAGGCAACGGTACAGGCTC |
| benR-top | GTCGTTGAGCCCTACAACGCTACCCTCTCGGTCCACCAGCTGGTCGAGAACTCTGACGcGACCTTCTGCATTGACAACGA |
|  |  |
| benR-bot | TCGTTGTCAATGCAGAAGGTCgCGTCAGAGTTCTCGACCAGCTGGTGGACCGAGAGGGTAGCGTTGTAGGGCTCAACGAC |
| TUB2-wt-top | GTCGTTGAGCCCTACAACGCTACCCTCTCGGTCCACCAGCTGGTCGAGAACTCTGACGAGACCTTCTGCATTGACAACGA |
| TUB2-wt-bot | TCGTTGTCAATGCAGAAGGTCTCGTCAGAGTTCTCGACCAGCTGGTGGACCGAGAGGGTAGCGTTGTAGGGCTCAACGAC |
| SURtop | GTATTCTCAGGACAGGTTGTTACCTCTGaTATTGGAAGCGACGCCTTCCAGGAGGCCGACGTCATAGGCATCTCCCGGTC |
| SURbot | GACCGGGAGATGCCTATGACGTCGGCCTCCTGGAAGGCGTCGCTTCCAATAtCAGAGGTAACAACCTGTCCTGAGAATAC |
| Sep5-ts-donor-top | GAAGGGGATTCAGTTCTGTCTGATGGTCTGTgagGCCTCAGGAACTGgtgagttGCAGGACTACCTTCGTCAACACTCTT |
| Sep5-ts-donor-bot | AAGAGTGTTGACGAAGGTAGTCCTGCaactcacCAGTTCCTGAGGCctcACAGACCATCAGACAGAACTGAATCCCCTTC  Primers used for generating PCR products to confirm mutations |
| PKS-ck-f | GGATTCCTCGCCGAGTTCTAC |
| PKS-ck-r | CAATCATCTCGGGGTTGTTGAGG |
| SEP6ts?-f | CACACCCTGAAGCCCCTTGATATC |
| SEP6ts?-R | CTCCTCGGTTGTGTGGATGAG |
